# New insights about the lymphatic vasculature in cardiovascular diseases

**DOI:** 10.12688/f1000research.20107.1

**Published:** 2019-10-29

**Authors:** Xiaolei Liu, Guillermo Oliver

**Affiliations:** 1 Center for Vascular and Developmental Biology, Feinberg Cardiovascular and Renal Research Institute, Feinberg School of Medicine, Northwestern University, Chicago, IL, 60611, USA

**Keywords:** lymphatics, lymphatic endothelial cells, cardiovascular diseases, myocardial infarction

## Abstract

The heart contains a complex network of blood and lymphatic vessels. The coronary blood vessels provide the cardiac tissue with oxygen and nutrients and have been the major focus of research for the past few decades. Cardiac lymphatic vessels, which consist of lymphatic capillaries and collecting lymphatic vessels covering all layers of the heart, transport excess fluid from the interstitium and play important roles in maintaining tissue fluid balance. Unlike for the coronary blood vessels, until a few years ago, not much information was available on the origin and function of the cardiac-associated lymphatic vasculature. A growing body of evidence indicates that cardiac lymphatic vessels (lymphatics) may serve as a therapeutic cardiovascular target.

## Introduction

Over the past few decades, the molecular characterization of the lymphatic vasculature, as well as a better understanding of its functional roles in pathophysiological conditions, has greatly improved ^1, 2^. As an important part of the circulatory system, the lymphatic vasculature is critical for the maintenance of fluid homeostasis, immune surveillance, and absorption of fat from the intestinal tract ^2, 3^. Original studies performed in sheep measured a flow rate of 1–5 ml/hour in both pre-nodal and post-nodal lymph under physiological conditions ^4^. However, lymph drainage can change greatly under disease conditions. Lymphatic malfunction has been related to a broad range of diseases including lymphedema, obesity, hypertension, and cancer ^1^. More recent findings argue that the lymphatic vasculature might also be functionally important in myocardial infarction (MI) ^5, 6^, congestive heart failure ^7^, obesity ^8, 9^, atherosclerosis ^10– 12^, and cardiac transplantation ^13^. Among these, heart failure is often a consequence of MI and remains the leading cause of morbidity and mortality in the Western world. Seeking therapeutic strategies is still among the top priorities in the cardiovascular field. Other than cardiovascular diseases, the emerging function of meningeal lymphatics in the clearance of cerebrospinal fluid (CSF) and drainage of interstitial fluid into the cervical lymph nodes has been another major recent finding related to novel lymphatic functional roles ^14– 17^. A recent article described that basal meningeal lymphatic vessels located in the lateral/basal part of the skull function as the main route for CSF ^17^. In this review, we discuss some of the recent findings about cardiac lymphatics in health and disease.

## The development and origins of cardiac lymphatics

Although lymphatic vessels were already described in the 17 ^th^ century, it was not until the late 20 ^th^ century with the identification of lymphatic endothelial cell (LEC) markers such as the transcription factor *Prox1* ^18^, the vascular endothelial growth factor receptor (VEGFR)-3 ^19^, integral membrane glycoprotein Podoplanin (Pdpn) ^20^, and the lymphatic vessel endothelial hyaluronan receptor 1 (LYVE1) ^21^ that our understanding of lymphatic development has drastically progressed. During embryonic development, lymphatics form after the blood vasculature. In mice, the first LECs appear at around embryonic day (E) 9.5, when a subset of venous endothelial cells start to express *Prox1* ^18^. *Prox1* is considered a master regulator of lymphatic development and LEC fate identity ^18^. In mice, these *Prox1*-positive LECs bud off from the cardinal vein and through migration and proliferation they eventually form the primitive lymphatic vascular network ^22^. By E14.5, a primitive lymphatic vasculature has developed in most embryonic organs, and at around that stage it undergoes remodeling to form a mature lymphatic network. In mammals, with the use of pig and dog models to track lymphatic flow, researchers reported several decades ago that lymphatics cover all layers of the heart: the subepicardium and myocardium ^23, 24^. More detailed analysis in mouse embryos reported that cardiac lymphatics form a few days after the development of the coronary blood vessels, with scattered Prox1 ^+^ and VEGFR-3 ^+^ LECs migrating from the ventricular surface of the sinus venosus such that lymphatics are detected on the dorsal epicardial surface of the heart at around E14.5 ^5^. During late embryonic development, LECs expand and cover the dorsal and ventral surfaces of the heart ventricles, and cardiac lymphatics are fully grown and mature by two weeks of age ^5^. Intriguingly, although most mammalian lymphatics are venous derived, lineage tracing suggested that a subset of cardiac LECs have a non-venous origin. A recent study using Isl1Cre reporter mice documented *Isl1*-expressing pharyngeal mesoderm progenitors as a potential non-venous origin of some cardiac lymphatics ^25^. Future studies should determine whether non-venous-derived cardiac lymphatics play functional roles under normal and pathological conditions.

## Emerging role of cardiac lymphatics in cardiovascular diseases

Similar to other organs, the heart relies on lymphatics to drain interstitial fluid to maintain homeostasis ^26^. Damage of cardiac lymphatics often caused by cardiac surgery leads to acute or chronic cardiac edema, infection, inflammation, and cardiac fibrosis ^27, 28^. Recent studies suggest that lymphatic growth plays beneficial roles in preventing or reducing cardiovascular diseases such as atherosclerosis ^10– 12, 29^ and MI ^5, 6, 30^. We will focus on some recent studies on lymphatics in these two conditions.

### Atherosclerosis

Atherosclerosis is a disease characterized by the overaccumulation of plaques which are made of fat, cholesterol, and immune cells inside the blood vessel wall. Over time, these plaques narrow and harden the arterial wall and eventually limit blood flow from the heart to the organs and other parts of the body ^31^. Thus, atherosclerosis is the leading cause of mortality worldwide and could lead to serious subsequent clinical outcomes including heart attack and stroke ^32^. Although the progression of this disease has not been completely understood, mobilization of cholesterol from the artery wall has been a solution to alleviate disease progression. It has only been recently that studies in mice showed that lymphatics are the main route for cholesterol transport to the bloodstream (a process termed reverse cholesterol transport, or RCT) ^10, 33^. Disruption of lymphatic function, either by surgical ablation of collecting lymphatic vessels or by using the *Chy* mice lacking functional dermal lymphatics, greatly impairs RCT ^10, 33, 34^. In contrast, induction of lymphangiogenesis by injection of VEGF-C, the ligand for VEGFR-3, into the mouse footpad decreased cholesterol content and improved RCT ^10^. Because VEGF-C also binds to VEGFR-2, a well-known receptor in blood endothelial cells that contributes to angiogenesis, administration of VEGF-C in this study cannot rule out contributions from the blood vasculature. More recently, another study injected a mutant form of VEGF-C (VEGF-C152S) that binds only to and activates signaling through VEGFR-3 but is unable to bind to VEGFR-2. The authors showed that treatment with VEGF-C152S promotes and maintains the rescue of the lymphatic dysfunction throughout the whole atherosclerotic process, restraining atherosclerotic plaque size and stabilizing plaque progression ^11^. In addition, a number of immune cell types, including macrophages and T and B cells, are thought to be involved in the development and progression of atherosclerosis ^35^. A recent study has shown that lymphatic capillaries are present in the adventitia of human and mouse atherosclerotic lesions and lymphatic vessel density is increased with plaque progression ^36^. Either blockage of lymphatic drainage or inhibition of VEGFR-3-dependent lymphangiogenesis aggravated atherosclerosis plaque formation, concomitantly with increased intimal and adventitial T cell density. These data suggest a beneficial role for adventitial lymphatics in plaque T cell accumulation in atherosclerosis ^36^.

### Myocardial infarction

MI, which is the most common heart injury, occurs by lack of blood supply to parts of the heart, leading to damage and rapid cardiomyocyte death. After MI, lymphatic vessel density increases robustly during the healing process, most likely to drain excessive fluid and to allow immune cell trafficking. However, the detailed characterization of the functional roles of cardiac lymphatics in pathological settings has been ignored until recently. Injection of recombinant human VEGF-C156S in the injured heart area after experimental MI resulted in increased lymphangiogenesis and improvement in cardiac function ^5^. Moreover, intramyocardial-targeted delivery of VEGF-C152S using microparticles as carriers accelerated lymphangiogenesis and improved myocardial fluid balance and attenuated cardiac inflammation, fibrosis, and cardiac dysfunction in a rat MI model ^6^. These studies indicated that therapeutic lymphangiogenesis could be a new approach for the treatment of heart diseases. However, it is still not clear whether the improved heart function is a direct consequence of increased cardiac lymphatics after MI. MI triggers a robust inflammatory response with mobilization of lymphocytes, neutrophils, and monocytes that help scavenge dead cells and release chemokines for cardiac remodeling ^6^. It is possible that VEGF-C therapy facilitates lymphangiogenesis and lymphatic function that in turn improves the resolution of cardiac edema and provides a pathway for inflammatory cell efflux, thus favoring wound healing within the injured heart. To further elucidate the mechanism by which VEGF-C-induced lymphangiogenesis improves cardiac function after MI, in a follow-up study, the authors documented a significant influx of circulating monocytes and activated macrophages that undertake extensive phagocytic activity in the infarcted region after MI; these immune cells in the injured heart are dependent on lymphatic vessels to circulate back to the lymph nodes ^37^. Therefore, stimulation of lymphangiogenesis by VEGF-C treatment after heart injury promotes the clearance of immune cells in the injured heart ^37^. Interestingly, this process is dependent on lymphatic endothelial LYVE1, as in *Lyve1 ^–/–^* mutant mice, immune cell trafficking and clearance to lymph nodes is blocked, resulting in the loss of viable myocardium, enhanced scarring, and significantly reduced cardiac output ^37^. This study suggests that therapeutic strategies to invoke lymphangiogenesis may prevent the inflammation-dependent progression to heart failure in acute MI patients. Another study investigated the role of VEGFR-3 in healthy hearts in response to ischemic injury by using sVEGFR-3 transgenic mice expressing a soluble decoy VEGFR-3 under the K14 promoter that blocks VEGFR-3 signaling and *Chy* mice, which have an inactivating mutation in VEGFR-3 ^38^. Blocking VEGFR-3 signaling did not affect cardiac function; however, after MI, sVEGFR-3 mice had significantly higher mortality with intramyocardial hemorrhages, a reduced capability to respond to lymphangiogenic signals, and a modified structure of the infarcted area. Interestingly, in this study, the authors did not observe differences in the inflammatory cell infiltration between different experimental groups, indicating that inflammatory activation is not altered by reduction of VEGFR-3. The high mortality in *Chy* and sVEGFR-3 mice after MI is possibly caused by the defective and leaky cardiac lymphatics, a consequence of VEGFR-3 downregulation ^38^. Other than VEGF-C/VEGFR-3 signaling, other signaling pathways and factors have been reported to improve cardiac functions after MI by regulating lymphangiogenesis. For example, adrenomedullin (AM) is a known cardioprotective peptide and has been previously reported to be essential for proper cardiovascular and lymphatic development in mice ^39^. Several pilot clinical studies reported that MI patients who received intravenous AM showed cardiovascular improvement ^40^. In a recent study, the authors showed that *Adm* (the gene that encodes the AM protein) overexpression in mice results in an increased number of lymphatic vessels post-MI compared to controls ^30^. Although *Adm ^hi/hi^* mice exhibit less cardiac edema and improved heart functions at 15 to 21 days post-MI, *Adm ^hi/hi^* male mice are delayed in resolving cardiac edema and heart functions compared to *Adm ^hi/hi^* females ^30^. AM regulates the gap junction protein connexin 43 in LECs ^41^. In the context of heart injury, overexpression of *Adm* increases gap junction coupling, improving heart functions and reducing cardiac edema after MI ^30^. Apelin is a bioactive peptide that plays a central role in angiogenesis and cardiac contractility ^42, 43^. Apelin promotes lymphatic development in zebrafish and pathological lymphangiogenesis in mice ^44, 45^. Functional inactivation of apelin in mice results in abnormal dilated and leaky lymphatics associated with a proinflammatory status after MI ^46^. Conversely, overexpression of apelin in ischemic hearts is sufficient to restore a functional lymphatic vasculature and reduce matrix remodeling and inflammation ^46^. Taken together, these studies documented that increased lymphangiogenesis improves heart function after cardiac injuries. However, whether increased lymphangiogenesis also improves lymphatic drainage functions is not yet known and will need to be further investigated. Nevertheless, these studies provide additional therapeutic strategies in the restoration of cardiac lymphatics to preserve cardiac functions.

### Lymphatic endothelial cells

During the last decade, many lineage tracing studies reported on the origins of LECs. Although it is still well accepted that the majority of mammalian LECs are venous derived, recent studies have reported non-venous-derived LECs in specific organ beds, especially in the heart. However, additional studies are needed to determine if these non-venous-derived LECs are functional during pathological settings. On the other hand, many of the present studies have highlighted that the restoration of lymphatic function or increased lymphangiogenesis might preserve a healthy cardiac microenvironment and cardiac homeostasis after MI or atherosclerosis. A better understanding of the molecular and functional mechanisms by which cardiac lymphatics participate in cardiovascular diseases could provide precise therapeutic strategies.

## Abbreviations

AM, adrenomedullin; LEC, lymphatic endothelial cell; LYVE1, lymphatic vessel endothelial hyaluronan receptor 1; MI, myocardial infarction; RCT, reverse cholesterol transport; VEGF, vascular endothelial growth factor; VEGFR, vascular endothelial growth factor receptor.
